# Identification of a Variable Number of Tandem Repeats Polymorphism and Characterization of LEF-1 Response Elements in the Promoter of the *IDO1* Gene

**DOI:** 10.1371/journal.pone.0025470

**Published:** 2011-09-27

**Authors:** Marion Soichot, Benjamin Hennart, Alaa Al Saabi, Audrey Leloire, Philippe Froguel, Claire Levy-Marchal, Odile Poulain-Godefroy, Delphine Allorge

**Affiliations:** 1 Equipe d'Accueil 4483, Faculté de Médecine de Lille, UDSL, Université Lille-Nord de France, Lille, France; 2 CNRS 8199 - Institute of Biology, Pasteur Institute, Lille 2 University, Lille, France; 3 Genomic Medicine, Imperial College London, Hammersmith Hospital, London, England; 4 Institut National de la Santé et de la Recherche Médicale, CIC EC 05, Hôpital Robert Debré, Paris, France; Florida State University, United States of America

## Abstract

**Background:**

Indoleamine 2,3-dioxygenase (IDO) catalyzes the first and rate-limiting step of the kynurenine pathway that is an important component of immunomodulatory and neuromodulatory processes. The *IDO1* gene is highly inducible by IFN-γ and TNF-α through interaction with *cis*-acting regulatory elements of the promoter region. Accordingly, functional polymorphisms in the *IDO1* promoter could partly explain the interindividual variability in IDO expression that has been previously documented.

**Methodology/Principal Findings:**

A PCR-sequencing strategy, applied to DNA samples from healthy Caucasians, allowed us to identify a VNTR polymorphism in the *IDO1* promoter, which correlates significantly with serum tryptophan concentration, controlled partially by IDO activity, in female subjects, but not in males. Although this VNTR does not appear to affect basal or cytokine-induced promoter activity in gene reporter assays, it contains novel *cis*-acting elements. Three putative LEF-1 binding sites, one being located within the VNTR repeat motif, were predicted *in silico* and confirmed by chromatin immunoprecipitation. Overexpression of LEF-1 in luciferase assays confirmed an interaction between LEF-1 and the predicted transcription factor binding sites, and modification of the LEF-1 core sequence within the VNTR repeat motif, by site-directed mutagenesis, resulted in an increase in promoter activity.

**Conclusions/Significance:**

The identification of a VNTR in the *IDO1* promoter revealed a *cis*-acting element interacting with the most downstream factor of the Wnt signaling pathway, suggesting novel mechanisms of regulation of *IDO1* expression. These data offer new insights, and suggest further studies, into the role of IDO in various pathological conditions, particularly in cancer where IDO and the Wnt pathway are strongly dysregulated.

## Introduction

The heme enzyme indoleamine 2,3-dioxygenase (IDO ; EC 1.13.11.52) catalyzes the initial and rate-limiting step of the kynurenine pathway that converts 95% of the essential amino acid tryptophan (Trp) to kynurenine derivatives. IDO is constitutively expressed in many cells and tissues and is also a cytokine-inducible enzyme, with IFN-gamma (IFN-γ) being the most potent known transcriptional inducer of IDO. The overexpression of IDO has a dual consequence: depleting Trp in local microenvironments and increasing the concentration of downstream metabolites of the kynurenine pathway.

IDO activation is of great interest in the field of immunology as a consequence of Trp starvation, as well as the generation of immunomodulatory kynurenine metabolites. It results in the inhibition of growth of various pathogens and, thus, participates in antimicrobial host defence mechanisms [1]. It has also a major immunosuppressive role by suppressing local T-cell responses [2], [3], thus preventing, for example, allogeneic fetal rejection during pregnancy [4], [5]. This immune suppression *via* IDO overexpression has also been shown to be involved in the immune escape of tumor cells which is a crucial feature of cancer progression [6]. As most of human tumors constitutively express IDO, this enzyme could be considered as a predictive marker in cancer progression and pharmacological IDO inhibition is currently regarded as a future strategy in cancer adjuvant immunotherapy [7]–[9].

Furthermore, as Trp is also involved in neurological functions as a precursor in the synthesis of serotonin, a neurotransmitter that modulates mood, cognition and several neuroendocrine rhythms [10], the kynurenine pathway appears to compete with the serotoninergic pathway for Trp availability. An increase in IDO activity consequently imbalances the kynurenine/serotonin pathways, thus resulting in a decrease of serotonin synthesis and an accumulation of kynurenine metabolites, most of them displaying neuroactive properties [11]. Accordingly, changes in peripheral and/or central serotonin and kynurenine metabolites concentrations have been shown to be involved in several neurodegenerative disorders, such as Alzheimer's and Parkinson's diseases, as well as in mental illnesses, such as schizophrenia and depression [11]–[13]. Additionally, IFN-alpha-induced activation of IDO is regarded as being responsible for neuropsychiatric side effects, such as anxiety and major depression, which can develop in patients chronically-treated by IFN-α immunotherapy, and trigger non-compliance or premature discontinuation of treatment [14], [15].

Although the role of IDO and its induction by cytokines are now well recognized, not many studies have focused on the mechanisms of variability of IDO activity, which could have numerous relevant clinical implications. *In vitro* studies have shown that the response of IDO to IFN-γ stimulation varies greatly between various human cell lines [16], [17]. Based on the kynurenine/tryptophan ratio, which is calculated from circulating concentrations and is recognized as a valid indicator of IDO activity [18], it appears that IDO activity exhibits relatively large interindividual variability, in particular in pathological conditions [19]–[24]. Indirect evidence of interindividual variability in IDO activity, and/or inductibility, is also supported by several studies that showed that the development of IFN-α-induced neuropsychiatric symptoms only occurs in 20–50% of cancer- or hepatitis C-treated patients [15], [25], [26].

The currently known causes of variation in IDO activity remain sparse. Physiological states such as ageing or pregnancy, as well as pathological conditions such as infection, inflammation or tumor proliferation, have been demonstrated to modify IDO expression and/or activity, but they all mainly reflect the various mechanisms involved in the regulation of IDO expression [13], [27]. However, it can be postulated that genetic factors could be involved in interindividual variability of IDO expression and/or activity. An indirect genetically-controlled mechanism of variation of expression is illustrated by a c.874T>A polymorphism that affects a NF-κB response element in the IFN-γ gene and, consequently, IFN-γ production, which in turn alters IDO up-regulation [28], [29]. Only one study, to our knowledge, has reported two rare naturally-occurring polymorphisms in the IDO-encoding gene (*IDO1*) which were shown *in vitro* to be responsible for impaired functional activity and could therefore result in reduced IDO activity in the general population [30]. However, in this study, only the 10 exons and the exon/intron junctions of *IDO1* were screened for nucleotide variations. As the promoter of *IDO1* carries numerous well-identified responsive element sequences that are involved in the regulation of IDO expression [17], [31]–[34], it prompted us to search for genetic polymorphisms in this functionally-crucial region of the *IDO1* gene. The screening of the *IDO1* promoter in healthy Caucasian subjects allowed us to identify a Variable Number of Tandem Repeats (VNTR) polymorphism. A significant association of this VNTR with decreased circulating tryptophan concentrations was observed in healthy female subjects. The contribution of the VNTR polymorphism to the variability of IDO expression was further studied by functional analyses. Using *in vitro* and *in silico* approaches, we revealed the existence of previously unknown *cis*-acting regulatory elements that could participate in transcriptional regulation and, therefore, in interindividual variability of *IDO1* expression.

## Results

### Identification of a functional VNTR polymorphism on the *IDO1* promoter

**Figure 1:**
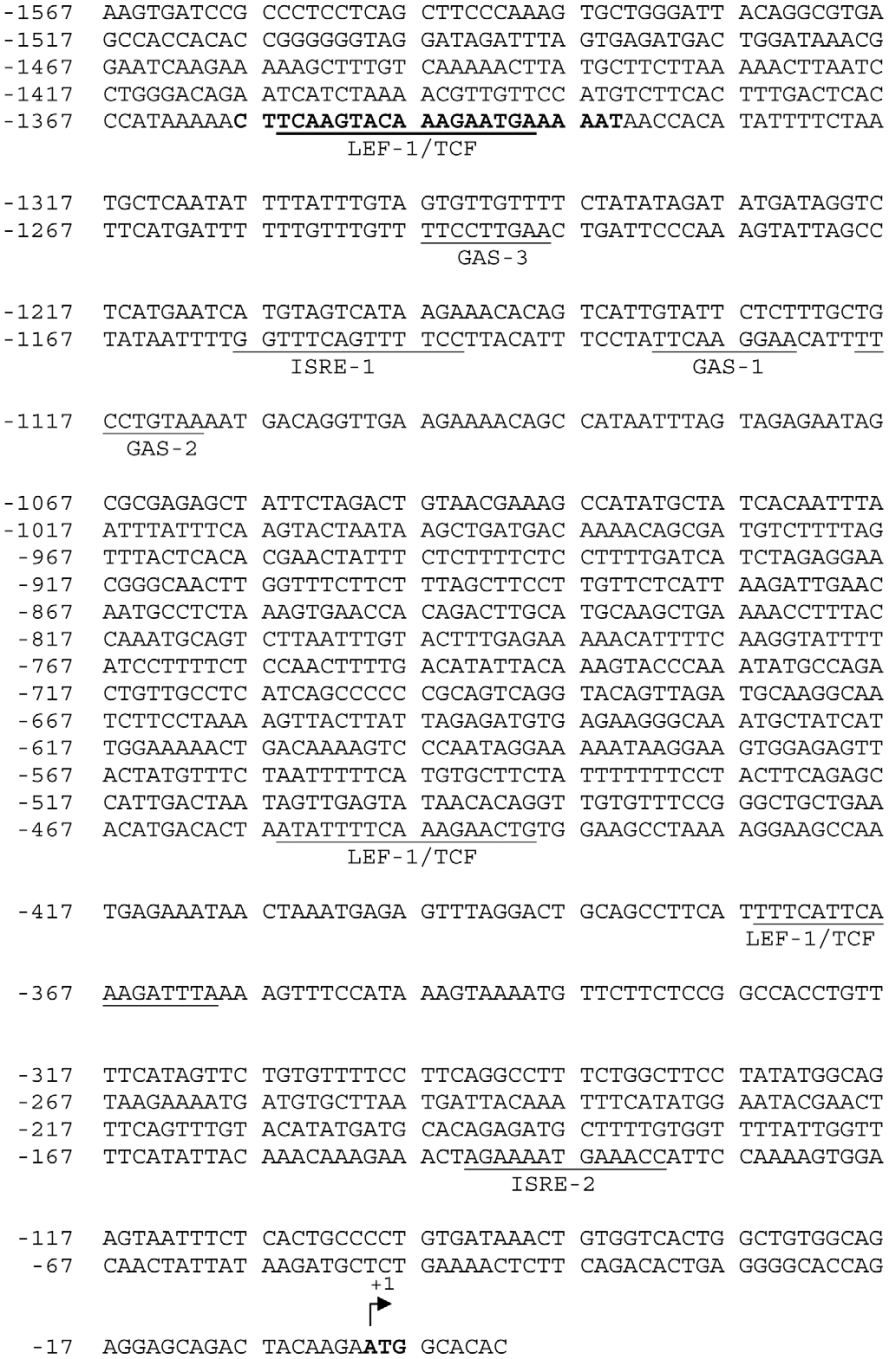
*Cis-acting* regulatory elements of the 1.6-kb promoter region of *IDO1*. Nucleotides ranging from -1567 upstream to 9 downstream of the translation initiation site (ATG) are shown. Transcription factor binding sites previously described (ISRE and GAS sites) or predicted by MatInspector (LEF-1/TCF sites) are underlined and named below the sequence. The VNTR motif is in bold letters.

To investigate potential sequence variations in the promoter region of *IDO1*, a PCR-sequencing strategy was developed and applied to a 1576 bp-sequence upstream of the ATG initiation codon. Sequencing was applied to PCR products amplified from genomic DNA samples of 41 unrelated healthy individuals of Caucasian origin. A VNTR polymorphism was identified in both heterozygous and homozygous states. This VNTR polymorphism comprises a 24-bp tandem repeat motif located approximately 1.3 kb upstream of the ATG initiation codon (Figure 1). This polymorphism is referenced as a deletion/insertion polymorphism with the RefSNP rs34820341 in NCBI. Here, we termed the two alleles containing one or two repeats,*V1 and *V2, respectively. The *V1 allele corresponds to the reference sequence of *IDO1* available in the GenBank database with the accession number NT_007995 and the sequence of the *V2 allele has been deposited in GenBank under the accession number JN382541. No other mutation was identified in the 1.6-kb promoter region of the 41 sequenced DNA samples.

The genotype and allele frequencies of the VNTR polymorphism in the 41 Caucasian volunteers, that were calculated based on the sequencing approach, are shown in Table 2. Using a rapid PCR-based genotyping assay, statistically similar genotype and allele frequencies were observed with the 300 DNA samples from the Haguenau cohort (Table 2). The frequency of the *V1 and *V2 alleles is around 46–48% and 52–54%, respectively, and the distribution of the VNTR genotypes respects the Hardy-Weinberg law.

**Table 1:** Primers used for *IDO1* promoter sequencing, VNTR genotyping, site-directed mutagenesis and post-ChIP PCR.

Primer designation	Primer sequence (5′→3′)^a^
**Primers for amplification and cloning** b **of the ** ***IDO1*** ** promoter (-1567 to -16)**	
IDOCLON-F	AA*GatATC*CGCCCTCCTCAGCTTC (-1567 to -1544)
IDOCLON-R	GTGTG*CCATgg*TTGTAGTCTGCTCC (-16 to +9)
**Primers for sequencing of the ** ***IDO1*** ** promoter**	
IDOPR-Seq1F	ATTCCCAAAGTATTAGCCTC (-1235 to -1216)
IDOPR-Seq2F	CAATGCCTCTAAAGTGAACC (-868 to -849)
IDOPR-Seq1R	CCATATAGGAAGCCAGAAAGGCC (-271 to -293)
IDOPR-Seq2R	GGTTACTTTAGAGGCATTG (-849 to -868)
**Primers for VNTR genotyping (-1421 to -1232)**	
IDOPR-F	AATCCTGGGACAGAATCATC (-1421 to -1402)
IDOPR-R	ATGAGGCTAATACTTTGGG (-1214 to -1232)
**Primers for site-directed mutagenesis** c	
IDOMUT-F	CCCATAAAAACTTCAAGTAC**G**A**C**GAATGAAAAATAACCAC (-1368 to -1329)
IDOMUT-R	GTGGTTATTTTTCATTC**G**T**C**GTACTTGAAGTTTTTATGGG (-1329 to -1368)
**Primers for amplification of immunoprecipitated target sequences**	
GAPDHPR-F	TACTAGCGGTTTTACGGGCG
GAPDHPR-R	TCGAACAGGAGGAGCAGAGAGCGA
IDOPR-site1-F	AATCCTGGGACAGAATCATC (-1421 to -1402)
IDOPR-site1-R	ATGAGGCTAATACTTTGGG (-1214 to -1232)
IDOPR-site2-F	CCTACTTCAGAGCCATTGAC (-530 to -511)
IDOPR-site2-R	GCAGTCCTAAACTCTCATTT (-386 to -405)
IDOPR-site3-F	AAATGAGAGTTTAGGACTGC (-405 to -386)
IDOPR-site3-R	CTTACTGCCATATAGGAAGC (-264 to -283)

F: forward; R: reverse

a Positions of the primers are based on the translational start site, with +1 corresponding to the adenine of the ATG codon.

b *Eco*RV and *Nco*I recognition site sequences (underlined nucleotides) were created in the primers for the cloning step of the *IDO1* promoter region.

c Bold letters represent the mutated nucleotides for site-directed mutagenesis.

In order to study the possible association between the VNTR genotype and IDO activity, tryptophan and kynurenine concentrations were measured in sera from 94 subjects of the Haguenau cohort, comprising 47 males (16 *V1/*V1, 16 *V1/*V2 and 15 *V2/*V2) and 47 females (15 *V1/*V1, 15 *V1/*V2 and 17 *V2/*V2). As serum samples were collected after an overnight fast, the effects of diet on circulating Trp levels can be considered as negligible. First, the complete sequencing of the 1.6-kb promoter region of *IDO1* excluded the presence of any other polymorphism in the 94 tested samples, and none of the samples carried the functional coding variants, i.e. the 9-bp deletion and/or the rs35099072 SNP. Mean (± S.D.) values of serum Trp (µM), serum Kyn (µM) and Kyn/Trp ratio (µmol/mmol), in all tested subjects, as well as in males and females separately, are reported according to VNTR genotypes in Table 3. Mean values (± S.D.) of each parameter are also shown independently of the VNTR genotypes. It could be observed that only the mean serum Trp concentration was significantly lower in females compared to males (54.9±13.6 µM *versus* 66.1±15.6 µM; p = 0.0006). In all subjects, no statistically significant association was observed between the VNTR genotype and serum Trp, serum Kyn or Kyn/Trp ratio. However, a lower Trp concentration was observed for females with a *V2/*V2 genotype compared to those with a *V1/*V1 (p = 0.076) or a *V1/*V2 (p = 0.028) genotype. Furthermore, the Trp concentration in females with a *V2/*V2 genotype was significantly lower than that in males with a *V1/*V1 (p = 0.008), *V1/*V2 (p = 0.01) and *V2/*V2 (p = 0.0007) genotype.

**Table 2:** Genotypic and allelic frequencies of the VNTR polymorphism in the *IDO1* promoter.

	Number of individuals			Number of individuals	
Genotype	N = 41^a^	n = 300^b^	Allele	n = 41^a^	n = 300^b^
*V1/*V1	22%	23.8%	*V1	46%	48.1%
*V1/*V2	49%	48.6%	*V2	54%	51.9%
*V2/*V2	29%	27.6%			

a The genotype and allele frequencies for the 41 DNA samples obtained from unrelated healthy Caucasian volunteers were calculated based on a sequencing approach, as described in the *Material and Methods* section.

b The genotype and allele frequencies for the 300 DNA samples obtained from the Haguenau cohort were calculated based on a PCR-based genotyping strategy, as described in the *Material and Methods* section.

### Effect of the VNTR polymorphism on the promoter activity of *IDO1*

**Figure 2:**
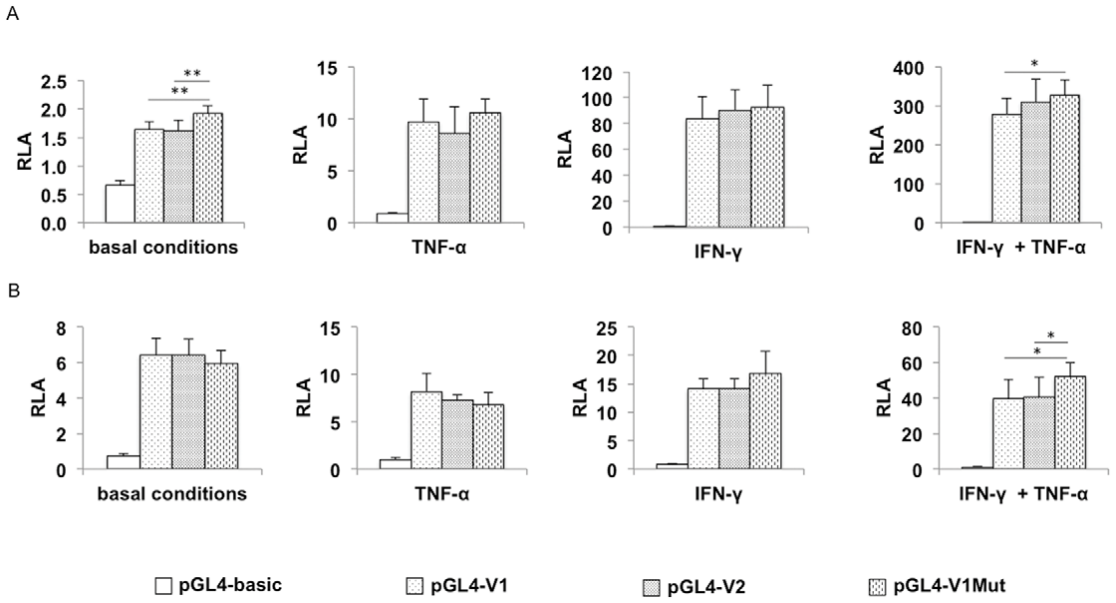
Functional analyses of *V1, *V2 and *V1Mut alleles of the *IDO1* promoter in HeLa cells. The four promoter constructs (pGL4-V1, pGL4-V2, pGL4-V1Mut and the promoterless pGL4-basic vector) and a *renilla* control vector (pGL4.74), without (A) or with (B) the VP16-TCF expression vector, were transiently transfected into HeLa cells. The luciferase activities were measured in basal conditions, or after stimulation by IFN-γ (10 ng/mL), TNF-α (5 ng /mL), or combined IFN-γ and TNF-α (10 ng/mL and 5 ng/mL, respectively). Results, expressed as relative *firefly* luciferase units normalized to *renilla* luciferase, are the mean ± SEM of triplicates from three independent experiments and are presented on a linear scale. Asterisks indicate statistical significance of * p<0.05 and ** p<0.001.

A gene reporter assay was developed in order to assess the impact of the VNTR on the transcriptional activity of the *IDO1* promoter. The luciferase activities shown in Figure 2A (see also Table S1) were measured under basal conditions or after stimulation by IFN-γ and/or TNF-α. Under basal conditions, the relative luciferase activities of the *V1 and *V2 alleles were increased 2.5-fold compared to the insertless promoter, which confirms that the 1.6-kb promoter region of *IDO1* has significant transcriptional activity. The transcriptional activity of the *IDO1* promoter was also evaluated after 24 h stimulation with IFN-γ and/or TNF-α, two cytokines that are known to induce *IDO1* expression. Stimulation by IFN-γ and TNF-α separately showed a respective 105- and 10-fold increase in luciferase activity of the *V1 and *V2 alleles compared to the pGL4 insertless vector (p<0.001). Stimulation in the presence of both IFN-γ and TNF-α resulted in a 250- and 277-fold increase in *V1 and *V2 luciferase activity, respectively (p<0.001 for both alleles). These data confirm the induction of *IDO*1 expression *via* a transcriptional mechanism through cytokines stimulation. Note, however, no statistical difference was observed between *V1 and *V2 luciferase activities in any of the above experimental conditions.

### Effect of the VNTR polymorphism on potential DNA-protein interactions

To assess whether the VNTR polymorphism could have an influence on upstream regulatory factors of the *IDO1* promoter, the 1.6-kb promoter region of *IDO1* was analysed using MatInspector prediction software. Three critical putative binding sites for the LEF-1/TCF transcription factor were identified in the *V1 allele, one site being located within the 24-bp motif of the VNTR, as shown in Figure 1. Consequently, the *V2 allele, which carries two repeat motifs of the VNTR, exhibits four potential TFBSs for LEF-1/TCF in the 1.6-kb promoter region.

**Figure 3:**
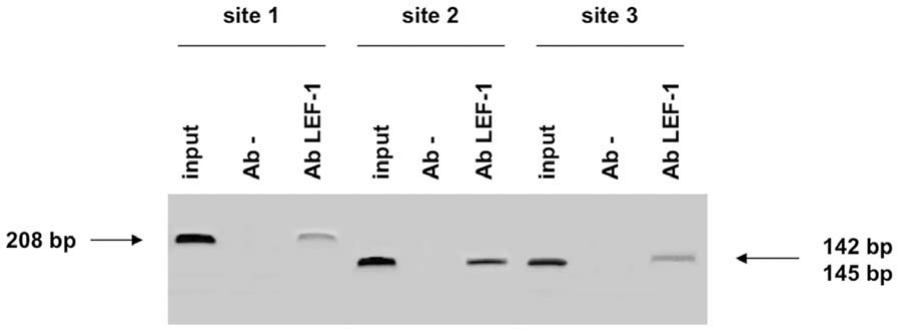
Binding of LEF-1 to the 1.6-kb *IDO1* promoter. PCR products were obtained from immunoprecipitates after ChIP assays of HeLa cells and loaded on a ethidium bromide stained-1% agarose gel. Three specific oligonucleotide primer pairs were used to confirm the presence of the three potential LEF-1/TCF binding sites on the 1.6 kb *IDO1* promoter region, as predicted by MatInspector. The three corresponding amplicons are sized 208 bp (site 1), 145 bp (site 2) and 142 bp (site 3). Positive controls (Input chromatin DNA before immunoprecipitation), negative controls without antibody (Ab-) and immunoprecipitation with anti-LEF-1 antibody are indicated for each amplified fragment of the *IDO1* promoter. Site 1 refers to the putative LEF-1 binding site within the 24-bp motif of the VNTR; sites 2 and 3 refer to the two additional LEF-1 sites predicted by MatInspector.

ChIP assays were used to confirm the presence of potential binding sites for LEF-1/TCF predicted by MatInspector. The assays were conducted with extracts of HeLa cells, that were genotyped as homozygotes for the *V1 allele, and the successful chromatin immunoprecipitation was checked *via* the GAPDH positive control. Three specific primer pairs were designed for PCR amplification of LEF-1 genomic sequences from immunoprecipitates.The results shown in Figure 3 indicate that LEF-1 binds to the three potential sequences in the *IDO1* promoter, which confirms the LEF-1/TCF binding sites predicted by MatInspector.

To evaluate the functional significance of the additional TFBS for LEF-1/TCF in the *V2 allele relative to *V1, gene reporter assays were conducted with the overexpression of an activated form of LEF-1. In this assay, activated LEF-1 is expressed due to the VP16-TCF expression vector possessing a virtual DNA-binding domain for TCF/LEF and a VP16 activation domain, in place of a *β*-catenin-binding domain [35]. For each situation, a negative control was conducted comprising an empty expression vector (pcDNA3.3). As previously, the promoter activity of both alleles was assessed in the presence and absence of cytokinic stimulation, and compared to the promoterless pGL4 vector (Figure 2B). With or without cytokinic stimulation, the luciferase activities of pGL4-V1 and pGL4-V2 were significantly enhanced in the presence of LEF-1 compared to co-transfection with the negative control (data not shown). Furthermore, the induction effect of IFN-γ and TNF-α and the synergistic effect of their association were also observed, as previously noted (Figure 2A/B). This second set of transfections showed that the presence of Wnt-activated LEF-1 increased the *IDO1* promoter activity for both *V1 and *V2 alleles. However, under the experimental conditions, no significant difference in promoter activity was observed between the two VNTR alleles.

### Effect of the LEF-1/TCF site located in the VNTR on *IDO1* promoter activity

To investigate the influence of the TFBS for LEF-1/TCF on the transcriptional activity of the 1.6-kb promoter region of *IDO1*, the *V1 allele was modified by mutating the core sequence of the binding site localized in the 24-bp motif of the VNTR by site-directed mutagenesis, such that the MatInspector software could no longer identify a binding site at this location. Thus, a third pGL4 construct containing the mutated core sequence of the LEF-1/TCF binding site (pGL4-V1Mut) was used in gene reporter assays, as described previously (Figure 2). As shown in Figure 2A, in basal conditions, the relative luciferase activity of pGL4-V1Mut was significantly increased compared to the control pGL4 vector, as well as compared to pGL4-V1 and pGL4-V2 constructs (p<0.001). Induction by IFN-γ or TNF-α increased the relative luciferase activity of pGL4-V1Mut 108- and 12-fold, respectively, with a synergistic effect of the combined cytokines resulting in a 292-fold increase, compared to that in basal conditions. However, the luciferase activities following IFN-γ or TNF-α stimulation were not statistically different between *V1, *V2 and *V1Mut alleles. Interestingly, the synergistic effect of the two cytokines resulted in a significantly higher relative luciferase activity for pGL4-V1Mut compared to that of pGL4-V1 (p<0.05).

Additional gene reporter assays conducted with the overexpression of an activated form of LEF-1 showed no statistical difference in the promoter activity of pGL4-V1Mut compared to pGL4-V1 and pGL4-V2, under basal conditions (Figure 2B). Furthermore, as in the absence of VP16-TCF, no significant differences were observed between the three constructs following a single stimulation with IFN-γ or TNF-α. However, the relative luciferase activity of pGL4-V1Mut was significantly higher following a combined cytokinic stimulation, with a 1.3-fold increase compared to pGL4-V1 and pGL4-V2 (p<0.05).

## Discussion

Variability in IDO activity can result in significant imbalances in the serotonin/kynurenine pathways which could have clinical immunological and neuropsychiatric implications. As inherited variants of coding and non-coding sequences of a gene are recognized as an important cause of interindividual variations in protein expression and/or activity, the identification of functional genetic polymorphisms in the *IDO1* gene would be of particular interest in association studies between IDO and various pathological conditions. Several variations in the *IDO1* genomic sequence have already been documented in sequence databases, but their functional consequences have not yet been studied [36]. In a recent study, Arefayene *et al*. [30] sequenced DNA samples of African-American and Caucasian origin and identified seventeen different polymorphisms in the 10 exons and intron-exon junctions of the *IDO1* gene. Two of these, a missense mutation (p.Arg77His; rs35099072) and a 9-bp deletion in exon 7, result, *in vitro,* in significantly reduced protein expression and, consequently, in almost complete loss of enzyme activity [36]. However, these functional variants appear to be very rare *IDO1* polymorphisms, since each was identified in only one African-American subject, and, accordingly, can be expected to have a minor phenotypic effect in the general population. In addition, Nishizawa *et al*. [37] studied *IDO1* genomic variations as a potential determinant of pre-eclampsia. The sequencing of placental genomic DNA from pre-eclamptic and normotensive pregnant women revealed three new rare *IDO1* variants, consisting of one silent mutation, one missense mutation (p.Arg193Gly) and a 4-bp deletion in the proximal 5′-UTR. However, no functional analyses were performed for the two coding SNPs, and the 4-bp deletion did not affect gene expression of *IDO1* in *in vitro* experiments [37].

As promoter regions control gene expression by interactions between *cis*-acting elements spanning through the promoter region and transcription factors, variations in the sequence of *cis*-acting elements could have an effect on gene expression [38]. In the case of *IDO1*, the promoter region has been well characterized and contains several *cis*-acting response elements that are involved in the up-regulation of the gene by cytokines, such as IFN-γ, the most potent inducer of IDO activity, as well as TNF-α [17], [32], [33]. Two Interferon-Stimulated Response Elements (ISRE) and three Gamma Activated Sequence (GAS) located within the 1300-bp upstream of the ATG initiation codon appear to be critical for maximal *IDO1* promoter activity, with a synergistic activation by IFN-γ and TNF-α [17], [32], [33]. In the present study, we identified a VNTR polymorphism in the *IDO1* promoter region, consisting of a 24-bp repeat motif located 1.3-kb upstream of the ATG initiation codon. It was identified by a PCR-sequencing strategy applied to 41 DNA samples and allowed us to characterize two different alleles, named *V1 and *V2, that carry one or two repeats, respectively, the *V1 allele corresponding to the reference sequence listed in Genbank (accession number NC_000008.10). This polymorphism appears to be common in Caucasians, the frequency of the *V1 allele being 46–48% and that of the *V2 variant being 52–54% in our study. No additional VNTR allele with more than two motif repeats was identified.

To assess the impact of the VNTR polymorphism on IDO activity, 47 males and 47 females from a cohort of 300 healthy Caucasian subjects were selected based on their genotype, and their sera were analysed to determine Trp and Kyn concentrations. We first observed a significant lower serum Trp concentration in females compared to males, as reported previously in other studies [39]–[41]. Furthermore, females with a *V2/*V2 genotype displayed a significant and a trend toward lower serum Trp concentration compared to females with a *V1/*V2 and *V1/*V1 genotype, respectively. IDO is known to be induced by soluble hormones, such as human chorionic gonadotropin, prolactin and estrogens, supporting the hypothesis of a hormonal control of IDO expression [42]–[45]. It can then be postulated that the VNTR polymorphism we identified has an effect on Trp metabolism under the influence of a female hormonal environment, which is partly supported by Carretti *et al*. [46] who showed that circulating Trp concentration has cyclic variations throughout the menstrual cycle.

In our experiments involving HeLa cells, a cell line which was previously used to study the mechanism of IFN-γ action on *IDO1* promoter activity [16], [32], luciferase reporter assays showed that the 1.6-kb fragment upstream of the translation start site is a functional promoter of *IDO1*, and that both IFN-γ and TNF-α induce promoter activity, a greater activation being obtained with IFN-γ and a synergy being observed with a combination of the two cytokines. However, no significant difference in promoter activity was seen between *V1 and *V2 alleles under any experimental condition. Based on these results, the absence of any difference in promoter activity between the two alleles, even after synergistic induction, may not have been unexpected, since the VNTR polymorphism does not modify any critical response element, such as ISRE or GAS.

A bioinformatic analysis was undertaken to predict potential *cis*-acting elements in the promoter region of *IDO1* in order to investigate whether the VNTR polymorphism could affect interactions between putative binding sites and transcription factors. In addition to the response elements for IFN-γ and TNF-α previously described, the *in silico* analysis predicted that the LEF-1/TCF transcription factor could interact with the promoter region of *IDO1* at three different sites, with one putative consensus sequence being located in the 24-bp motif of the VNTR. As TFBS prediction programs cannot assert the existence of a TFBS or infer the functionality of a site [47], additional experiments were undertaken. Initially, the presence of the three potential binding sites for LEF-1 in the *IDO1* promoter was confirmed by specific PCR amplifications using chromatin immunoprecipitation of HeLa cellular extracts. To investigate the functionality of the potential sites, additional gene reporter assays were performed using an overexpression of a functional LEF-1. As a member of the TCF/LEF family of transcription factors, LEF-1 is one of the major nuclear transducers involved in Wnt signaling. However, it has no transcriptional activation potential in isolation and requires binding with *β*-catenin, the *β*-catenin activation domain and TCF/LEF DNA-binding domain forming a bipartite transcriptional activator of target genes [48]. VP16-TCF is a plasmid construct that expresses a form of LEF-1 fused to the potent VP16 transactivation domain which is constitutively active and obviates the need for a *β*-catenin TCF/LEF interaction [35], [49]. In the presence of VP16-TCF, the promoter activity of the *V1 and *V2 alleles was found to be significantly increased relative to the negative control expression vector, thus confirming the existence of specific and functional binding sites for LEF-1 on the studied 1.6-kb promoter region of *IDO1.* This result confirms the reliability of MatInspector prediction of potential binding sites for this transcription factor. However, no difference in promoter activity was observed between *V1 and *V2 alleles, thus suggesting the insertion of the 24-bp motif, that creates one additional copy of a LEF-1 response element, may not interact with LEF-1, and, furthermore, that the other three LEF-1 sites most likely contribute to the global increase of *IDO1* promoter activity following overexpression of LEF-1. Interestingly, following IFN-γ and/or TNF-α stimulation, promoter activities of *V1 and *V2 alleles were similarly increased, but to a lesser extent than those observed in the first experiments without LEF-1 overexpression, relative to the promoterless pGL4 vector. These results suggest the interaction between LEF-1 response elements and VP16-TCF product alters the *IDO1* promoter responsiveness to cytokines. It is noteworthy that Robinson *et al.* [33] showed previously that the deletion of a large sequence between the two ISRE sites in the *IDO1* promoter gave rise to a significant increase of transcription activation by IFN-γ, compared to that observed with the undeleted promoter. Accordingly, the authors suggested that regulatory elements within this region could provide additional mechanisms of *IDO1* regulation. As two of the LEF-1 sites we characterized are located between the two ISRE sites, they could then be hypothesized to correspond to those suggested additional *cis-acting* elements in the *IDO1* promoter.

The modification, by site-directed mutagenesis, of the LEF-1 binding site located within the 24-bp motif of the VNTR, appeared to increase *IDO1* promoter activity relative to the activity of the *V1 and *V2 alleles, suggesting that the LEF-1 site within the VNTR has a repressive effect on *IDO1* promoter activity. This result could be explained by the fact that LEF-1 can also interact with proteins of the Groucho family, proteins that are known to repress transcriptional activation by *β*-catenin-TCF complexes [48], [50]–[52]. However, the effect of the pGL4-V1mut construct on luciferase activities appears rather marginal compared to that of cytokine stimulation.

In conclusion, we have characterized a previously unknown VNTR polymorphism in the promoter region of the *IDO1* gene. Although this VNTR appears to have no major consequence on basal or cytokine-induced transcriptional activity *in vitro*, it seems to participate in Trp homeostasis in female subjects. Furthermore, the identification of this polymorphism has uncovered previously unknown *cis*-acting elements for the LEF-1 transcription factor. Variations in the number of these response elements could alter *IDO1* expression, particularly in pathological conditions involving the Wnt signaling pathway.

## Materials and Methods

### Biological samples and ethics statement

41 anonymous DNA samples, previously obtained from unrelated healthy Caucasian volunteers and declared to the Commission Nationale de l′Informatique et des Libertés for research studies on genetic polymorphism identification (SPFCO/cellule de bioéthique/n° DC-2008-642), were used to screen the genomic sequence of the *IDO1* promoter.

A further 300 serum and DNA samples from young healthy adults, prepared as previously described by Bouatia-Naji *et al*. [53], were selected from the Haguenau cohort whose study was approved by the ethics committee of Paris-St Louis University, Paris, France [54]. All experiments were approved by the IRB at Paris-St Louis University, Paris, France (permit number: protocole 2006/05-C05-57). The Haguenau cohort had been fully described previously [54]. Briefly, this cohort included subjects selected between 1971 and 1985 from a population-based registry in the city of Haguenau, France, who were initially classified in two sub-groups based on birth data. At 22.1±3.9 years of age, participants gave blood samples for serum and DNA analyses in order to study predisposition to various metabolic disorders. From these serum and DNA samples, we randomly selected 300 samples obtained from subjects who were born with appropriate gestational age and presented no metabolic disorder at adult age. The serum samples were collected after an overnight fast.

A written informed consent was obtained from all participants.

### Sequencing of the *IDO1* promoter region

The promoter region of the *IDO1* gene from each of the initial 41 DNA samples was sequenced in order to screen for genetic polymorphisms. Accordingly, a DNA fragment covering the 1576 bp-sequence upstream of the ATG initiation codon was amplified from each DNA sample using Phusion™ High-Fidelity DNA polymerase (Ozyme, Saint-Quentin-en-Yvelines, France), according to the manufacturer's instructions. Each fragment was directly sequenced using the BigDye® Terminator v3.1 cycle sequencing kit (Applied Biosystems, Courtaboeuf, France) and an automated 3130XL genetic analyzer (Applied Biosystems). All primers were designed according to the nucleotide sequence of the *IDO1* gene published in GenBank (accession number NT_007995) and are listed in Table 1.

**Table 3:** Mean serum concentrations (± S.D.) of tryptophan (Trp), kynurenine (Kyn) and Kyn/Trp ratio in 94 healthy Caucasian subjects according to their *IDO1* VNTR genotype.

	All subjects			Males			Females		
	(n = 94)			(n = 47)			(n = 47)		
	*V1/*V1	*V1/*V2	*V2/*V2	*V1/*V1	*V1/*V2	*V2/*V2	*V1/*V1	*V1/*V2	*V2/*V2
	(31)	(31)	(32)	(16)	(16)	(15)	(15)	(15)	(17)
Trp	60.5±15.6			66.1±15.5			55.0±13.6^a^		
(µM)	62.3±16.1	59.1±11.2	60.2±18.7	66.4±17.3	60.4±11.8	71.8±15.9	57.9±14.0	57.7±10.6^b^	50.0±14.9c,d
Kyn	2.1±0.9			2.2±0.9			2.1±0.9		
(µM)	2.1±1.1	2.2±0.8	2.1±0.9	2.1±1.1	2.2±0.8	2.4±0.8	2.2±1.0	2.3±0.9	1.8±0.9
Kyn/Trp	35.4±13.5			33.4±11.1			37.4±15.4		
(µmol/mmol)	34.5±17.5	37.6±11.4	34.2±10.7	30.6±13.3	35.6±10.6	34.0±8.9	38.7±20.9	39.7±12.3	34.4±12.3

a p<0.001 when comparing females and males.

b p<0.05 when comparing females *V1/*V2 and males *V2/*V2.

c p<0.05 when comparing females *V2/*V2 and females *V1/*V2.

d p<0.01 when comparing females *V2/*V2 and each subgroup of males, *V1/*V1, *V1/*V2 and *V2/*V2.

### Genotyping assay for VNTR identification

A rapid and simple genotyping assay was developed to genotype the VNTR polymorphism that was identified within the *IDO1* promoter region and applied to the 300 DNA samples from the Haguenau cohort. A short PCR fragment encompassing the VNTR region was amplified with a pair of specific primers (Table 1). The PCR reaction was carried out in a final volume of 25 µL using Taq DNA polymerase (Invitrogen, Cergy Pontoise, France) and the amplicons separated on 10% acrylamide gel and visualized using UV transillumination after ethidium bromide-staining. The discrimination between a 208-bp fragment (*V1 allele) and a 232-bp fragment (*V2 allele) allowed the characterization of the VNTR genotype of each sample.

### Genotyping assays for two rare polymorphisms in the coding sequence of *IDO1*

Additional *IDO1* genotyping assays were performed on 94 out of the 300 DNA samples from the Haguenau cohort to screen the 9-bp deletion and the rs35099072 SNP, previously characterized as functional *IDO1* variants by Arefayene *et al.* [30]. The corresponding PCR and sequencing conditions are available on request.

### Bioinformatics analysis

The prediction software MatInspector (www.Genomatix.de) was used to search the *IDO1* gene for potential Transcription Factor Binding Sites (TFBSs) in the 1.6-kb promoter region. A restrictive threshold of 0.85 and the matrix library v7.0 were used.

### Plasmid constructs and site-directed mutagenesis

Genomic DNA samples carrying a homozygous VNTR genotype, i.e. *V1/*V1 or *V2/*V2, were used to amplify a 1576-bp or 1600-bp fragment, respectively, using a pair of primers that were previously used to sequence the *IDO1* promoter (Table 1). These primers allow the amplification of a PCR fragment with *Eco*RV and *Nco*I sites at the 5′- and 3′-ends, respectively. Following amplification and purification, the fragments were double-digested by *Eco*RV and *Nco*I endonucleases (Gentaur, Paris, France) and subsequently cloned into the pGL4.11 [Luc2P] vector (Promega, Charbonnières, France) which contains the *firefly* luciferase cDNA. After cloning, both recombinant vectors pGL4-V1 and pGL4-V2 were sequence-verified to avoid constructs containing sequence mismatches and correct orientation confirmed.

The LEF-1/TCF consensus sequence identified in the repeat motif of the VNTR by MatInspector was mutated using the QuickChange II Site Directed Mutagenesis Kit (Agilent Technologies, Massy, France), according to the manufacturer's recommendations. This mutagenesis step was applied to the pGL4-V1 construct with complementary primers designed to create a 2-bp change in the LEF-1/TCF core sequence. The corresponding plasmid was named pGL4-V1Mut and was used for gene reporter assays. The sequences of the mutated primers are shown in Table 1.

### Transient transfection and luciferase assays

The HeLa human cervical carcinoma cell line (LGC Standards, Molsheim, France) was maintained and cultured in DMEM supplemented with 1% of a combined solution of 100 U/mL penicillin and 100 µg/mL streptomycin and 10% FBS (Invitrogen). The cells were grown in 5% CO_2_ at 37°C. The day before transfection, 4.10^4^ cells were freshly plated on 24-well plates in 0.5 mL of complete media. Twenty-four hours after plating, HeLa cells were transfected with 1.25 µL of lipofectamine LTX (Invitrogen).

In a first set of transfection experiments, HeLa cells were transfected with 250 ng of the insertless pGL4.11-basic vector or one of the recombinant vectors, pGL4-V1, pGL4-V2 and pGL4-V1Mut, and 12.5 ng of the control reporter PGL4.74 plasmid (Promega) that contains the *renilla* luciferase, used to normalize transfection efficiency. Four hours after transfection, the medium was removed and replaced with fresh complete medium with or without IFN-γ (10 ng/mL), TNF-α (5 ng/mL), or a combination of both cytokines (Sigma-Aldrich, Saint Quentin-Fallavier, France). Luciferase activities were measured 24 h after cytokine induction.

In a second set of transfections, 125 ng of the pGL4.11 basic vector or one of the promoter constructs was co-transfected with 12.5 ng of PGL4.74 and 125 ng of the expression vector VP16-TCF; pcDNA 3.3 was used as a negative expression control. As previously, a cytokine induction was performed 4 h after transfection and luciferase activities were measured 24 h later.

The luciferase activities were measured using the Dual-Glo Luciferase Reporter Assay System (Promega) following the manufacturer's recommendations. The *firefly* luciferase activity (fLA) and the *renilla* luciferase activity (rLA) were measured sequentially with a Glomax luminometer (Promega). The relative luciferase activity (RLA) was calculated as follows: RLA  =  fLA/rLA. The transfection and reporter gene assays were performed in triplicates and repeated in three independent experiments. Results are expressed as relative *firefly* luciferase units normalized to *renilla* luciferase. Data are expressed as mean ± SEM.

### Chromatin Immunoprecipitation

ChIP analysis was performed according to the Upstate Biotechnology (Millipore, Molsheim, France) protocol with modifications. 3.10^6^ HeLa cells were cross-linked for 10 min at 37°C in 1% formaldehyde. Cross-linking was stopped by adding 0.125 M of glycine for 5 min at room temperature. Cells were then washed twice with cold PBS 1X, re-suspended in 300 µL of SDS lysis buffer containing protease inhibitors and pelleted for 5 min at 2000 g at 4°C. Cross-linked DNA was sheared by sonication to yield DNA fragments between 200 and 500 bp in size and then diluted 10-fold with ChIP dilution buffer. At this step, 20 µL of the sample was used as input material to quantify the amount of DNA present in each sample before immunoprecipitation. Samples were pre-cleared for 4 h with 75 µL of protein A-agarose/salmon sperm DNA (50% slurry) and immunoprecipitated overnight at 4°C with rotation with 10 µg of an antibody to acetylated histone H3 (Millipore) for positive control experiments, 20 µg of an anti-LEF-1 antibody (Tebu Bio, Le Perray en Yvelines, France), or without antibody as a negative control.. The following day, 60 µL of protein A-agarose/salmon sperm DNA (50% slurry) was added to the sample for 4 h at 4°C with agitation to collect the antibody/histone complex. Beads were then collected by gentle centrifugation and washed according to the Upstate protocol. The histone complexes were eluted from the beads with 500 µL elution buffer (1% SDS, 0.1 M NaHCO3) at room temperature for 30 min with rotation. Cross-links were reversed by adding 20 µL of NaCl 5M to the eluates and heating at 65°C overnight. The sample was then treated with 10 µL of 0.5 M EDTA, 20 µL of 1 M Tris-HCl, pH 6.5, and 2 µL of 10 mg/mL Proteinase K for 2 h at 45°C. DNA was recovered by phenol/chloroform extraction and ethanol precipitation. PCR used to isolate each of the 3 potential TFBSs for LEF-1, as well as the GAPDH promoter sequence used as a positive control, were performed with specific primers listed in Table 1 and amplicons were loaded on a ethidium bromide stained-1% agarose gel.

### Determination of serum concentrations of tryptophan and kynurenine

Trp and kynurenine (Kyn) concentrations in serum samples from 47 male (mean age, 29.3±4.5 years) and 47 female (mean age, 29.4±4.2 years) healthy subjects of the Haguenau cohort, that comprise three groups of 31, 31 and 32 subjects with a *V1/*V1, *V1/*V2 and *V2/*V2 genotype, respectively, were determined by HPLC/MS-MS using a method described by Schefold *et al.* [55], with slight modifications. One hundred µL of serum was analysed after addition of 50 µL methanol containing methyl-clonazepam (Sigma-Aldrich, Saint-Quentin Fallavier, France) at 1.25 mg/L as an internal standard (IS); and 50 µL acetonitrile. The samples were mixed and then centrifuged (11200 g, 6 min). One hundred µL supernatant was added to 500 µL deionised water and centrifuged at 11200 g for 3 min. 15 µL were injected into an UPLC/MS-MS system (Acquity TQ Detector, Waters, Milford, MA) equipped with an electrospray ion source and a HSS C18 column (1.8 µm – 2.1 X 150 mm, Waters). A positive ion mode was used in order to detect ions, using multiple reaction monitoring. Nitrogen was the collision gas. Flow solvent was a gradient of acetonitrile and water containing formic acid (0.01%), for a total run time of 5 min per sample. Quantitative precursor ions and qualitative product ions selected for measurements of Trp, Kyn and IS were 205.1–146.0, 209.0-94.1 and 330.1–255.1, respectively. Calibration curves for quantification were established with spiked samples of human albumin and correlated with the concentration of IS. Data were analysed using Chromalynx Software (Waters). The Kyn/Trp (µmol/mmol) ratio, an indicator of IDO activity, was calculated by relating concentration of Kyn (µM) to Trp (µM).

### Statistical analysis

Observed genotype frequencies of the VNTR polymorphism were examined for deviation from the Hardy-Weinberg equilibrium by χ^2^ testing. The luciferase assay data were tested using a non-parametric Mann-Whitney test (two-tailed). The above statistical calculations were run with the SPSS software version 15.0 (SPSS Inc., Chicago, IL). Serum Trp, serum Kyn and Kyn/Trp ratio were each compared with genotype frequencies in all subjects, as well as in males and females separately, using a non-parametric Mann-Whitney test (two-tailed) with use of GraphPad Prism version 5.02 for Windows. Findings were considered statistically significant at p value<0.05.

## Supporting Information

Table S1 **Mean (**± **S.E.M) luciferase activity values observed with pGL4-basic, pGL4-V1, pGL4-V2 and pGL4-V1mut constructs in HeLa cells.**
(DOC)
