# Supplementary material for: Identification of a Variable Number of Tandem Repeats Polymorphism and Characterization of LEF-1 Response Elements in the Promoter of the IDO1 Gene
Source: PLoS One. 2011 Sep 27;6(9):e25470. doi: 10.1371/journal.pone.0025470 (PMC3181322; doi:10.1371/journal.pone.0025470)
Supplement: Table S1 — Mean (± S.E.M) luciferase activity values observed with pGL4-basic, pGL4-V1, pGL4-V2 and pGL4-V1mut constructs in HeLa cells. (DOC) [file pone.0025470.s001.doc]

| **Constructs** | **Basal conditions** | **TNF-α**  **(5 ng/mL)** | **IFN-γ**  **(10 ng/mL)** | **IFN-γ (10 ng/mL)**  **+ TNF-α (5 ng/mL)** |
| --- | --- | --- | --- | --- |
|  | **Without LEF-1 overexpression** | | | |
| **pGL4-basic** | 0.66 ± 0.09 | 0.88 ± 0.11 | 0.85 ± 0.16 | 1.12 ± 0.15 |
| **pGL4-V1** | 1.64 ± 0.13 | 9.71 ± 2.24 | 84.02 ± 16.64 | 279.10 ± 40.76 |
| **pGL4-V2** | 1.61 ± 0.19 | 8.61 ± 2.54 | 90.54 ± 15.82 | 310.35 ± 57.52 |
| **pGL4-V1Mut** | 1.93 ± 0.12 | 10.61 ± 1.31 | 92.35 ± 17.43 | 327.38 ± 40.07 |
|  | **With LEF-1 overexpression** | | | |
| **pGL4-basic** | 0.74 ± 0.14 | 1.00 ± 0.23 | 0.80 ± 0.16 | 1.02 ± 0.33 |
| **pGL4-V1** | 6.43 ± 0.92 | 8.18 ± 1.88 | 14.12 ± 1.81 | 39.58 ± 10.79 |
| **pGL4-V2** | 6.42 ± 0.91 | 7.26 ± 0.58 | 14.13 ± 1.78 | 40.53 ± 11.24 |
| **pGL4-V1Mut** | 5.93 ± 0.73 | 6.77 ± 1.32 | 16.77 ± 3.96 | 51.90 ± 7.83 |
